# Latent Cognitive Profiles Predict One-Year Health Outcomes in Older Adults Without Dementia

**DOI:** 10.1093/geroni/igaf122.1961

**Published:** 2025-12-31

**Authors:** Junxin Li, Jiaying Li

**Affiliations:** Johns Hopkins University, Baltimore, Maryland, United States; Johns Hopkins University, Baltimore, Maryland, United States

## Abstract

Traditional classifications of cognitive status often overlook domain-specific cognitive deficits in older adults. This study aims to identify distinct cognitive profiles in older adults without dementia and evaluates their one-year predictive value for key health outcomes, including falls, sleep difficulties, depressive symptoms, and daily functioning. We analyzed data from 2,219 adults aged ≥65 years in the National Health and Aging Trends Study. Baseline cognitive performance, assessed across six domains at Round (R) 12 (with supplemental data from R11), included episodic memory, executive function, orientation, psychomotor function, visual attention, and working memory. Latent profile analysis identified five cognitive subgroups, and logistic regression models examined associations with one-year outcomes at R13, excluding pre-existing events.The five profiles were: (1) Overall intact (50.5%); (2) Isolated moderate orientation impairment (15.6%); (3) Mild global impairment with preserved orientation (22.0%); (4) Mild global impairment with significant orientation impairment (5.5%); (5) Moderate global impairment (6.2%).At one-year follow-up, compared to Profile 1, Profile 5 had higher odds of falls (OR 1.98), Profile 4 was linked to prolonged sleep onset (OR 2.04), and Profiles 4 and 5 had higher odds of depressive symptoms (OR 2.55–2.69). Functional difficulties were observed in Profile 2 (shopping: OR 1.79; banking: OR 2.84) and Profile 5 (medication management: OR 2.98; laundry: OR 2.11; banking: OR 3.42). Nearly half of dementia-free older adults exhibit distinct cognitive profiles with varying risks for key outcomes. These findings highlight the need for domain-specific cognitive assessment to guide targeted interventions that support independent living and aging in place.

